# Behaviour of Low Molecular Weight Compounds, Iron and Copper of Wine Spirit Aged with Chestnut Staves under Different Levels of Micro-Oxygenation

**DOI:** 10.3390/molecules25225266

**Published:** 2020-11-12

**Authors:** Sara Canas, Florina Danalache, Ofélia Anjos, Tiago A. Fernandes, Ilda Caldeira, Nádia Santos, Laurent Fargeton, Benjamin Boissier, Sofia Catarino

**Affiliations:** 1 Instituto Nacional de Investigação Agrária e Veterinária, Quinta de Almoinha, Pólo de Dois Portos, 2565-191 Dois Portos, Portugal; florina.danalache@iniav.pt (F.D.); ilda.caldeira@iniav.pt (I.C.); 2 MED–Mediterranean Institute for Agriculture, Environment and Development, Instituto de formação avançada, Universidade de Évora, Pólo da Mitra, Ap. 94, 7006-554 Évora, Portugal; 3 Instituto Politécnico de Castelo Branco, Quinta da Senhora de Mércules, 6001-909 Castelo Branco, Portugal; ofelia@ipcb.pt; 4 CEF, Instituto Superior de Agronomia, Universidade de Lisboa, Tapada da Ajuda, 1349-017 Lisboa, Portugal; 5 Centro de Biotecnologia de Plantas da Beira Interior, Quinta da Senhora de Mércules, 6001-909 Castelo Branco, Portugal; 6 CQE, Centro de Química Estrutural, Associação do Instituto Superior Técnico para a Investigação e Desenvolvimento (IST-ID), Universidade de Lisboa, 1049-001 Lisboa, Portugal; tiago.a.fernandes@ist.utl.pt; 7 Adega Cooperativa da Lourinhã, Av. de Moçambique, 2530-111 Lourinhã, Portugal; adega.lourinha@gmail.com; 8 Vivelys, Domaine du Chapître, 34750 Villeneuve-les-Maguelone, France; laurent.fargeton@vivelys.com (L.F.); benjamin.boissier@vivelys.com (B.B.); 9 LEAF—Linking Landscape, Environment, Agriculture and Food Research Center, Instituto Superior de Agronomia, Universidade de Lisboa, Tapada da Ajuda, 1349-017 Lisboa, Portugal; sofiacatarino@isa.ulisboa.pt; 10 CEFEMA-Center of Physics and Engineering of Advanced Materials, Instituto Superior Técnico, Universidade de Lisboa, Av. Rovisco Pais, 1, 1049-001 Lisboa, Portugal

**Keywords:** phenolics, furanic aldehydes, iron, copper, wine spirit, ageing, micro-oxygenation, chestnut wood, heat maps

## Abstract

Alternative technologies for a more sustainable wine spirits’ ageing have been studied but a lack of knowledge on the effect of oxygenation level remains. This work examined the behaviour of low molecular weight compounds, iron and copper of a wine spirit aged in 50 L demijohns with chestnut wood staves combined with three levels of micro-oxygenation or nitrogen. Compounds and mineral elements were quantified by HPLC and FAAS, respectively, in samples collected at 8, 21, 60, 180, 270 and 365 days of ageing. Results showed that most of the compounds underwent significant changes in their content over time and behave differently depending on the wine spirit’s oxygenation level: higher contents of gallic acid, syringic acid and vanillin were associated with lower micro-oxygenation level while higher contents of ellagic acid, syringaldehyde, coniferaldehyde and sinapaldehyde resulted from higher one; lowest contents of these compounds were found in the nitrogen modality. Weak correlation between copper and the studied compounds was evidenced whereas closer relationship between iron, vanillin, gallic, syringic and ellagic acids at end of ageing was observed. This study provides innovative information on the role of oxygen in wine spirit’s ageing, and on chestnut wood effect on wine spirit’s mineral composition.

## 1. Introduction

**Figure 1:**
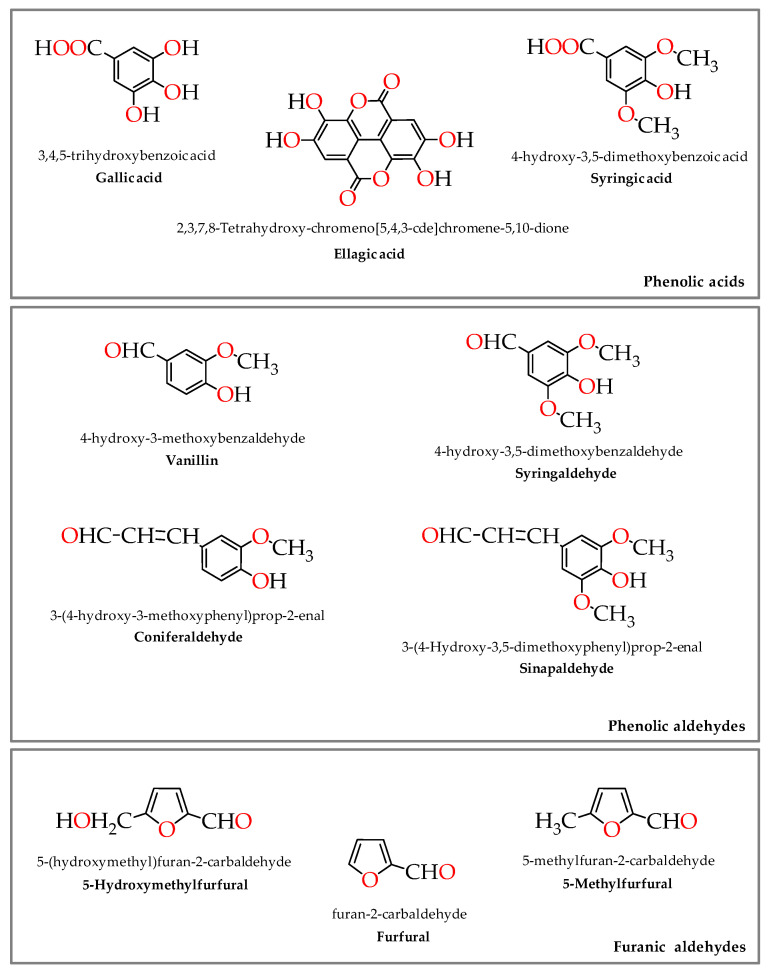
Chemical structure of phenolic acids, phenolic aldehydes and furanic aldehydes present in the aged WS.

Research made over the last twenty years revealed a close relationship between the low molecular weight compounds released from the wood into the wine spirit (WS) and the sensory properties acquired during the ageing process, which are responsible for its quality [1,2]. Despite the matrix complexity and the puzzling mechanisms involved in the connection stimuli–sensations perceived by the sense organs and the human brain [3], positive correlations have been found between such compounds (Figure 1) and the colour [4], the flavour [5], the taste and the mouthfeel [6] of spirit drinks. Among them, the relation between furanic aldehydes—‘dried fruits’ and ‘caramel’ flavours, and especially between vanillin—‘vanilla’ flavour [5], are of utmost importance for the aged WS distinctiveness. Specifically, ‘vanilla’ is one of the most appreciated and popular flavours in the world, and seems to act as a favourable sensory property to consumer’s preference for foods and beverages [7]. In addition, some of these wood-derived phenolic compounds impart antioxidant activity to the aged WS [8], thus making it as a contributor to a phenolic rich diet, with higher added value. Indeed, the ingestion of bioactive compounds, through a moderate consumption of this spirit drink, can have a positive health effect [9], as opposed to ethanol-induced damage [10].

These phenolic compounds and furanic aldehydes found in the aged WS result from the wood itself and/or from its processing in cooperage through the degradation of wood biopolymers (lignin, cellulose and hemicelluloses) and tannins [2]. Indeed, the wine distillate is devoid of phenolic compounds except some volatile phenols, and of furanic aldehydes except furfural [11]. For this reason, the ageing stage plays a crucial role in refining the beverage. The changes underlying ageing are consequences of several physicochemical phenomena (of additive and subtractive nature) involving the wood and the wine distillate, particularly the extraction of wood compounds and the oxidation reactions [2].The extraction and the set of factors ruling it have been extensively studied [2], however few is known about the oxidation that occurs during the WS ageing and the involved compounds [12,13]. This is believed to be a key topic to understand the ageing chemistry and, therefore, to better explore the ageing technology, as it has been done for wine [14,15], towards the improvement of the ageing process and of the final product.

**Figure 2:**
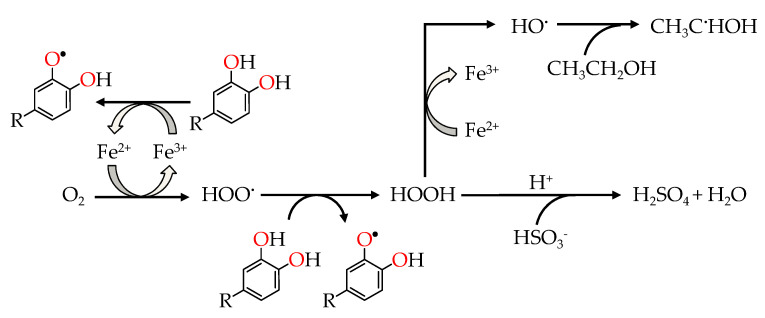
Fe-catalysed wine oxidation scheme [25].

Furthermore, mineral elements extracted from the wood [16,17] and existing in the spirit drinks [18,19,20,21,22], such as iron (Fe) and copper (Cu), seem to play an essential role on such oxidation reactions. Oxygen does not react directly with phenolic compounds without the presence of transition metals, which catalyse these processes due to their ability to readily donate and accept electrons (redox cycle) [14,23,24]. The limitation of the reactivity of molecular oxygen (triplet state, O_2_ cannot form bonds by accepting electron pairs) is overcome by the stepwise addition of a single electron, which can be provided by reduced transition metal ions, essentially ferrous (Fe^2+^) and cuprous (Cu^+^). More precisely, the sequential electron transfer reduces molecular oxygen to hydroperoxide radical (–HOO^•^) and hydrogen peroxide (H_2_O_2_). H_2_O_2_ is expected to react with Fe^2+^ or Cu^+^ ion (the Fenton reaction) to give rise a hydroxyl radical (–HO^•^) capable of oxidizing almost any organic molecule in the WS, as in the wine, e.g., ethanol to acetaldehyde [14,25]—Figure 2.

**Figure 3:**
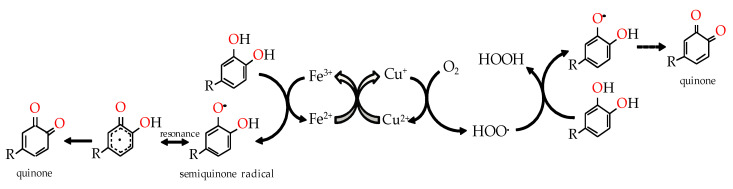
Catalytic action of iron and copper ions in the oxidation of catechols for the generation of quinones and hydrogen peroxide.

Studies made on wine showed that, as the primary substrates for oxygen, phenolic compounds are the major targets of hydroperoxyl radicals [25]. In particular, phenolic compounds containing a catechol or a galloyl group, such as (+)-catechin, (−)-epicatechin, gallocatechin, gallic acid and its esters, and caffeic acid are sequentially oxidized to by-products (*ortho*-semiquinone radicals and benzoquinones), generically named as quinones, while oxygen is reduced to hydroperoxyl radicals (–HOO^•^ and –HO^•^) and H_2_O_2_, and the whole process is mediated by a synergistic effect of Fe^3+^/Fe^2+^ and Cu^2+^/Cu^+^ redox cycle [14,23,26]—Figure 3. Compounds containing 1,2,3-trihydroxyl, *ortho*-dihydroxy aromatic ring or *para*-dihydroxyl aromatic ring are more readily oxidized because the resultant phenoxyl semiquinone radical can be stabilized by a second oxygen atom. The catechol functional groups tend to be the main reacting species with hydroperoxyl radical and, when reacting, to form the semiquinone radical and hydrogen peroxide. Oxygen is converted to the next oxidation state by the donation of a hydrogen radical to the hydroperoxyl radical, which forms hydrogen peroxide.

The intervention of Fe, Cu and manganese (Mn) ions in oxidative processes of other main substrates for oxidation in wine, e.g., ethanol, ascorbic acid and tartaric acid, had been reported [24,27]. In particular, Mn and Fe play a primary role in chemical processes with acetaldehyde in wine by favouring acetaldehyde formation (Mn) and catalysing its combination with phenolic compounds (Fe) [26,28]. According to Cacho et al. [27], the higher the Fe concentration the lower the acetaldehyde content (due to its combination with phenolic compounds as it is being formed) and higher the polymerization of phenolic compounds by acetaldehyde. Also, a relation between radical formation and Cu content was observed in sugar cane spirits, even if the exact nature of radical formed by ageing was not identified [29].

WS contains both high levels of substrates for oxidation, namely ethanol and phenolic compounds extracted and/or derived from the wood, and metals with important catalytic role. Fe content in spirit drinks is normally lower than 1 mg/L, while Cu concentrations frequently range between 1 and 3 mg/L [18,19,20,21,22,30,31]. Having in mind that wood ash contains Fe and Cu besides other elements [32], the release of these metals to the WS during ageing is expected. However, extremely limited data are available regarding the effect of wood on the elemental composition of WS and other distillates during ageing, and to the best of our knowledge no data is available for chestnut wood.

In this context, the wood species and the oxygen are decisive factors. Studies made on different kinds of wood corroborated that chestnut wood (*C. sativa* Mill.) stands out from oak wood (*Q. robur* L., *Q. petraea* (Matt.) Liebl., *Q. pyrenaica* Willd., *Q. alba* L.) by the high quality aged WSs and faster ageing afforded, which is ascribed to the high pool of extractable compounds and specific anatomical features of the former [33,34]. Regarding oxygen, in the traditional ageing, using wooden barrels, transfer is made through the wood and through the space between staves [35]. Although the high-quality WSs obtained, the high cost and length of this process has led to search for alternatives. An alternative ageing technology for WS, replicating the traditional one, using wood pieces combined with micro-oxygenation (MOX) in stainless steel tanks is under development [4]. Despite the promising results attained, a single flow rate of oxygen (2 mL/L/month) was applied. In order to optimise such technology towards maximization of WS quality and ageing sustainability, further investigation is being carried out under the Project Oxyrebrand (https://projects.iniav.pt/oxyrebrand/index.php/pt/). For this purpose, the same Lourinhã wine distillate was aged in pilot scale, using 50 L glass demijohns, with chestnut wood staves inside combined with three levels of MOX or nitrogen, with two replicates. It is intended to understand the reactions involved, particularly the oxidation ones, and the possible role of iron and copper as catalysts on such reactions.

Hence, in the present work the behaviour of target compounds (gallic acid, ellagic acid, syringic acid, vanillin, syringaldehyde, coniferaldehyde, sinapaldehyde, furfural, 5-hydroxymethylfurfural and 5-methylfurfural) quantified by HPLC, together with that of iron and copper, quantified by FAAS, under different oxygenation levels during 365 days of ageing, was examined.

## 2. Results and Discussion

### 2.1. Individual Behaviour of Mineral Elements and Low Molecular Weight Compounds

The wine distillate used to fill the demijohns, which was analysed at the beginning of the experiment (0 days), had 0.086 mg/L of iron and 0.939 mg/L of copper (Table 1) and was devoid of the quantified low molecular weight compounds other than furfural (4.3 mg/L) (Tables 2–4).

Regarding the results obtained during the ageing period with different levels of MOX, it should be noted that the information of Table 5 (see Section 3.1) is pivotal for their interpretation, taking into account that: (i) in the first 15 days of ageing, the oxygen flow rate was the same in O15, O30 and O60 modalities (2 mL/L/month); (ii) from 15 to 30 days, it decreased in O15 (to 0.6 mL/L/month) and remained in O30 and O60 (2 mL/L/month); (iii) from 30 to 60 dias, it decreased in O30 (to 0.6 mL/L/month) and remained in O60 (2 mL/L/month); (iv) from 60 days on, the oxygen flow rate was the same in O15, O30 and O60 (0.6 mL/L/month).

For this reason, in the first sampling time (8 days), the contents of mineral elements and compounds studied were not significantly different because the aged WSs were under the same MOX level. Little variations in the content of each analyte between MOX modalities were due to the wood variability (even with the same surface to volume ratio used) and the variability of MOX flow rate, among others. In the second sampling time (21 days), the differences between the aged WSs are mainly ascribed to the lower level of oxygen supplied to O15. In the third (60 days) and following sampling times, the differences between the aged WSs are assigned to the three levels of MOX applied.

On the other hand, taking into account the experimental design (see Section 3.1), the comparison between the MOX modalities and the control one (N) was examined from 8 days on.

#### 2.1.1. Iron and Copper

Both Fe and Cu contents are of primary importance for WS quality. As aforementioned, Fe concentration in this product and similar distillates is normally lower than 1 mg/L, being mainly introduced during the ageing process by the contact with metallic surfaces and release from the wood [21]. Cu concentrations often range between 1 and 3 mg/L in wine distillates obtained from distillation devices made of copper (column still and alembic). Indeed, copper surfaces can release Cu to a significant extent (increasing with contact time and alcohol strength), which is entrained through the condensation of the steam along the compartments of the distillation apparatus [36]. In spite of that, this metal is still used in distillation devices due to its malleability, heat conductor capacity, and resistance to corrosion.

The one-way ANOVA results for the contents of total iron and copper in the WSs during the 365 days of ageing are shown in Table 1. They are presented in a table together with the corresponding graphics to clearly show the significance level of the differences found and to better elucidate the behaviour of these mineral elements according to the ageing modalities (O15, O30, O60 and N) and the ageing time (8, 21, 60, 180, 270 and 365 days).

The concentrations of total Fe found in WSs were quite low, varying between 0.086 and 0.28 mg/L, and in accordance with the normal variation range [18,19,30,31]. At each sampling time, the Fe content of the WS was not significantly influenced by the ageing modality.

Time had a significant impact on its concentration, with slight and progressive increases until 270 days of ageing. This enrichment, although not relevant from the technological point of view, is most probably explained by Fe release from the wood into the WS throughout ageing. This hypothesis is supported by previous works on the effect of wood ageing on wine mineral composition, carried out both with oak barrels and oak staves [16,17], in which Fe enrichemnts were observed. In a very recent study on the release of wood extractable elements in experimental spirit model using different wood species, the variability of this element content according to the specific botanical origin was evident [37]. However, the literature lacks information with respect to chestnut wood. From 270 to 365 days a significant decrease was observed in the WS from all ageing modalities.

**Figure 4:**
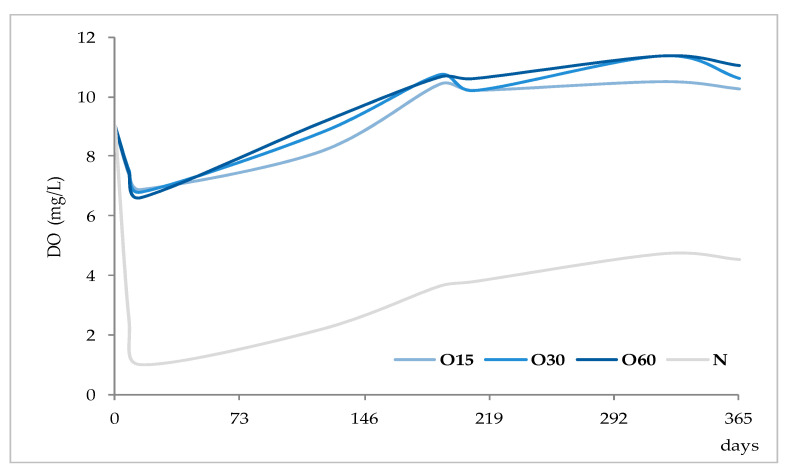
Average dissolved oxygen content in the WSs during the ageing period (measurement made on a water-based basis).

Depending on the oxidation-reduction potential of the medium, metal ions can be present under different oxidation forms in different proportions, decisively influencing insolubilisation and precipitation phenomena. According to studies made on wine, Fe is present in both Fe^2+^ and Fe^3+^ ion forms, being partially involved in soluble complexes with organic acids. Ferric iron [Fe(III)] is more likely to form complexes than ferrous iron [Fe(II)]. Fe(III) and Fe(II), in both ions and complexes, constitute total Fe. It is well known that wine is more susceptible to ferric haze and precipitation after aeration, as this increases the proportion of Fe^3+^ form responsible for such phenomenon [38]. More specifically, ferric iron is known to react with phenolic compounds of red wine producing a soluble complex that later floculates and precipitates. Furthermore, the interaction between tannic acid (500 mg/L) with Fe^2+^ was shown [39]. Therefore, it is quite predictable that a similar phenomenon may have occurred in WS, causing Fe precipitations due to reactions with phenolic compounds, namely hydrolysable tannins, released from the wood. This hypothesis is supported by the evolution of dissolved oxygen over time (with the highest concentrations being observed between 270 and 365 days) (Figure 4) and by the kinetics of phenolic compounds in the WSs (presented and discussed below), both favouring this phenomenon.

Regarding Cu, the concentrations were low, ranging from 0.939 to 0.320 mg/L, and in accordance with the values reported in the literature [18,19,30,31]. Although no significant effect of ageing modality was observed, Cu concentrations tended to be higher in the WS obtained with higher supply of oxygen. This is likely due to the different oxidation-reduction potential of the medium, governing the balance of Cu oxidation forms (Cu^2+^ and Cu^+^), thus influencing solubilisation and precipitation phenomena. Indeed, Cu insolubilisation occurs in wine when the oxidation-reduction potential reaches an appropriately low level for Cu^2+^ reduction to Cu^+^, unlike ferric haze [38,40].

The ageing time had a significant impact on Cu concentrations in WS but, unlike Fe, progressive decrease was observed until 180 days. The depletion of this metal is positive from the WS quality perspective, given its potential participation in physicochemical instability phenomena and the potential risk to WS safety [19,20,21]. Between 180 and 270 days no significant differences were observed. From 270 to 365 days a slight increase was noticed. The depletion during the first months of ageing can be assigned to the insolubilisation and precipitation of Cu. Previous studies [41] reported the impact of this mineral element in turbidity and precipitation phenomena of irreversible nature in WS, at pH higher than 4.5., e.g., through the formation of copper tannate. Moreover, this hypothesis is supported by the evolution of pH over the time, from 5.33 in wine distillate at the beginning of the experiment to values close to 4.20 in all the WSs at 365 days. Lastly, the slight increase of total Cu concentration observed between 270 and 365 days suggests Cu extraction and release from the wood, which is supported by previous studies on the topic [17,37] although focused on other wood botanical species. Despite the high amount of phenolic compounds in the WS available to react with Cu, probably the pH value lower than 4.5 did not favour the formation of copper tannate, since binding mode to Cu^2+^ depends on the pH of the solution, the ratio of metal to ligand and the medium in which the complex formation occurs [40].

#### 2.1.2. Phenolic Acids

Phenolic acids are non-flavonoid compounds found in the aged WS as a result of the wood contact. The toasted wood (oak and chestnut) commonly used in enology comprises hydroxybenzoic acids (C_6_-C_1_), such as gallic acid and syringic acid, as well as ellagic acid, a dilactone that has origin on the hydrolytic release of 6,6’-dicarbonyl-2,2′,3,3′,4,4′-hexahydroxybiphenyl units (HHDP) from ellagitannins [42]. They exist in the wood in the free form or conjugated, and also derive from the thermal degradation of gallotannins, lignin and ellagitannins, respectively, during the heat treatment in cooperage [43].

The one-way ANOVA results for the WS’ phenolic acids contents during the 365 days of ageing are shown in Table 2. They are presented in a table together with the corresponding graphics for the above-mentioned reason for Table 1.

From a general perspective, the results of Table 2 show that the aged WSs had higher content of gallic acid than other phenolic acids, namely ellagic acid, confirming the findings of previous works on chestnut wood, either in barrels [33] or in alternative technology using staves and MOX [4]. According to these works, gallic acid can act as a chemical marker for this kind of wood and for the corresponding aged WSs.

##### Gallic Acid

At each sampling time, the gallic acid content of the WS was not significantly influenced by the ageing modality (Table 2). However, at 21, 60, 180, 270 and 365 days of ageing, higher content of this acid (45–123 mg/L) was found in the WS in which the lowest level of oxygen was applied (O15). Intermediated (38–106 mg/L) and lower (33–95 mg/L) gallic acid contents, although of the same order of magnitude, were exhibit by the WSs submitted to an intermediate level (O30) and higher level of oxygen applied (O60), respectively. Similar effect was noticed by Castellari et al. [44] in red wine stored for six months in stainless steel tanks with two levels of oxygen supplied. Finally, the WS from the control modality (N) had the lowest content throughout the ageing period (16–86 mg/L).

The results obtained over the time reveal a significant increase of gallic acid between 21 and 180 days in all WSs; higher increment occurred in O15, which was aged under mild oxidative conditions. From 180 to 365 days, a non-significant variation was observed in all modalities, with a trend to stabilize (O15) or to decrease (O30 and O60).

The content found in each WS reflect the balance between additive phenomena, which contribute to increase the level of free gallic acid, such as the extraction from the wood and the gallotannins’ degradation in the liquid medium [45], and subtractive phenomena, responsible for decreasing the level of free acid, such as the oxidation reactions and interactions with other compounds in the liquid medium. As aforementioned, gallic acid is prone to oxidation with the formation of quinones. Since these reaction products are highly reactive, they can further react with nucleophilic compounds. Hence, the oxidation of gallic acid, which is one of the most abundant low molecular weight compounds of the aged WS, can trigger a cascade of oxidation and other reactions, involving phenolics and other compounds. According to the literature on WS [12], esterification, acetalization, polymerisation and condensation reactions occur subsequent to the oxidation of phenolic compounds, for which catalysts may be required (iron and copper in the free or complex form).

Therefore, the lower level of oxygen applied (O15) to the WS may have favoured the extraction of gallic acid from the wood but limited its subsequent oxidation and further reactions. Higher levels of applied oxygen (O30 and O60) may have promoted additive phenomena but, to a greater extent, subtractive phenomena (in which iron may have been involved), causing lower concentration of gallic acid throughout ageing.

The lowest content of this acid found in the control modality (N), in which the dissolved oxygen content was decreased as much as possible with the nitrogen supply, reinforces the central role played by oxygen in the evolution of gallic acid during ageing. It is noteworthy that gallic acid content was not null in this ageing modality, as all the compounds studied, because the extraction from the wood can slowly occur without the external addition of oxygen [46]; some oxygen contained in the wood itself is released into the WS (estimated at 8.67 mg per 100 g of staves [15]), mostly in the first month of ageing [47].

Lastly, a collateral aspect should be addressed. Chestnut wood is rich in gallic acid, which undergoes oxidation in the presence of small amounts of oxygen, supplied through MOX or through the barrel, and creates favourable conditions to chemical reactions involving other wood-derived compounds and/or those of the distillate. Thus, this feature, together with the highest pool of extractable compounds and specific anatomical features [33], allows explaining the faster evolution of WS aged with this kind of wood than with oak wood, and the specific sensory properties and antioxidant activity imparted.

##### Ellagic Acid

Table 2 shows that the ellagic acid content of the WSs was only significantly different, according to the ageing modality, at 270 days of ageing: the MOX modalities (O15, O30, O60) induced higher content than the control one (N). Despite the lack of significance between the MOX modalities, the ellagic acid content remained slightly higher in the WS with greater oxygenation (O60) after 60 days of ageing.

Besides, the analysis of the ellagic acid content in each ageing modality over the time unveils its significant increase in the WS O60 between 60 and 270 days, followed by a significant decrease (9.5–23 mg/L). The same statistical outcome was found for the WS O30, which had a slightly lower level of this acid (8.6–22 mg/L). Similar behaviour was observed in the WS under lower MOX level (O15) from 21 to 270 days, followed by a downward trend (4.5 to 23 mg/L). After 21 days, the control WS (N) presented the lowest content of ellagic acid (4.3–19 mg/L), with greater gap from the others after 60 days of ageing.The greatest increment of ellagic acid until 60 days of ageing mainly reflected its extraction from the wood to the WS (additive phenomenon), due to a higher concentration gradient [46]. At the same time, ellagitannins are also transferred from the wood to the liquid medium and give rise to ellagic acid [48]. According to Navarro et al. [49], oxygen is a core element in these phenomena; García-Estéves et al. [47] found that the oxygen contained in the wood plays an important role on them. This last aspect explains the evolution of ellagic acid in the control WS (N). In turn, the sharp increase of ellagic acid concentration recorded between 180 and 270 days, particularly in the WSs under MOX effect, was likely due to other additive phenomena: the oxidation and hydrolysis of ellagitannins in the liquid medium [45,47,48,50]. According to studies made on model solutions [47,51], the ellagitannins reach their maximum concentration in the aged beverage in the first months of ageing, and easily react with oxygen. However, different ellagitannins show different reactivity towards oxygen [47,50] due to specific redox potential, or steric effects, which is closely related to their chemical structure [51]. This feature may have ruled their disappearance/degradation over the ageing time and therefore the release of ellagic acid in the WS. The works conducted by García-Estévez and collaborators [47,50] on model-solutions with wood chips under controlled oxygenation revealed that vescalagin, grandinin and roburin E are more involved in reactions with oxygen in the beginning of ageing, while castalagin reacts later. In addition, the higher the dissolved oxygen level the higher the amounts and rate of ellagitannins disappearance/degradation as a consequence of oxidation reactions, as well as reactions that do not involve oxygen directly (such as hydrolysis), and reactions boosted by the products of ellagitannins oxidation that can react with the wood-derived ellagitannins [47,50]. Hence, these reactions seemed to be more intense in the WS from O60 modality, contributing to a greater accumulation of ellagic acid. The decrease of this compound in all modalities at the end of ageing, indicates that the oxidation reactions and the subsequent ones (subtractive phenomena) were favoured by the higher levels of dissolved oxygen in these WSs (Figure 4), surpassing the additive phenomena; taking into account the kinetics of iron in this period, it is plausible to assume its involvement in such reactions.

Moreover, considering the high positive correlation between ellagic acid and the WS’ antioxidant activity [8], the variation of its content with the MOX modality may influence this feature of the studied WSs.

##### Syringic Acid

The results obtained in each sampling time (Table 2) show that the syringic acid content of the WSs was significantly affected by the oxygen level at 180 and 365 days of ageing, based on the difference between WSs in which oxygen was applied (O15, O30, O60) and the control one (N), which exhibited lower content. Although no significant differences were observed between the MOX modality, the WS from O15 presented slightly higher content between 21 and 365 days than the WSs from O30 and O60.

These results suggest that the syringic acid accumulation was favoured by a less oxidative environment during ageing (O15), which may have hindered oxidation and other subtractive phenomena (namely esterification, giving rise to ethyl syringate [13] and other esters, and complexation with iron [52]), but a certain level of oxygen was needed to promote extraction of this compound from the wood and other additive phenomena. Indeed, syringic acid can result from the oxidation of syringaldehyde [53], which is in the free form in the aged WS as a consequence of direct extraction from the wood [46] and lignin’s hydroalcoholysis in this oxidative and acidic medium [53]. Acetic acid is the main responsible for this last condition as the most plentiful acid of the aged WS. This acid exists in the wine distillate and its content also increases over the ageing stage [54] as a result of wood extraction (from hemicelluloses) and oxidation of acetaldehyde [55]. Research made on wine [14,25] showed that acetaldehyde may arise from the ethanol oxidation triggered by the oxidation of phenolic compounds.

Interestingly, the syringic acid content of the WSs O30 and O60 showed a regular and significant increase between 60 and 365 days (4.8–12 mg/L and 5–12 mg/L, respectively). In the WS O15 there was a gradual and significant increase in concentration between 21 and 180 days, followed by a resembling concentration until 270 days, and then a further significant increase (3.1–13 mg/L). Since the behaviour between 180 and 270 days was observed in the WSs from the two replicates of O15 modality, a problem in the sampling of the spirits should be excluded. Therefore, the resembling concentration can be ascribed to temporary conditions that balanced additive and subtractive phenomena. An insufficient level of oxygen in this WS for the oxidation of syringaldehyde (whose content increased in the same period—Table 3) but also for the oxidation of the syringic acid itself and/or for its involvement in other reactions might have happened.

The lowest content of syringic acid was found in the control WS (N) throughout ageing, ranging from 1.4 to 9 mg/L, with greater distance from the others after 60 days of ageing. This evolution should be closely related to the availability of oxygen provided by the wood as aforementioned.

#### 2.1.3. Phenolic Aldehydes

Phenolic aldehydes are non-flavonoid compounds usually classified into two groups: (i) the benzoic aldehydes (C_6_-C_1_), which can be monomethoxylated (guaiacyl-type; vanillin) or dimethoxylated (syringyl-type; syringaldehyde); (ii) the cinnamic aldehydes (C_6_-C_3_)_,_ monomethoxylated (guaiacyl-type; coniferaldehyde) and dimethoxylated (syringyl-type; sinapaldehyde). These compounds are found in the toasted wood [33], from the wood itself [42], in which they exist either in the free form or linked to the cell wall constituents, and may also arise from lignin breakdown during the heat treatment in cooperage [56], being released into the WS during ageing.

The one-way ANOVA results for the WSs’ phenolic aldehydes contents during the 365 days of ageing are displayed in Table 3. They show that higher contents of syringyl-type aldehydes (sinapaldehyde followed by syringaldehyde) than guaiacyl-type aldehydes (coniferaldehyde followed by vanillin) were found in the studied WSs.

##### Vanillin

Comparing the WSs from the different ageing modalities in each sampling time (Table 3), a significant effect of the oxygen supplied on the vanillin content was only observed at 270 days. This differentiation was based on a greater enrichment of vanillin in the WS from the MOX modalities (O15, O30, O60) than in the control one (N) which content varied between 0.66 and 4.7 mg/L. However, there was no consistent behaviour over this period in any of the modalities subject to MOX: slightly higher content in O15 at 21 days, 60, 270 and 365 days; slightly higher content in O60 at 180 days. 

Examining the evolution of vanillin concentration over the time, different kinetics were observed depending on the ageing modality: (i) the intermediate oxygen level applied (O30) induced a progressive and significant increase of vanillin in the WS between 60 and 365 days (2.5–5.7 mg/L); (ii) the lowest oxygen level supplied (O15) promoted a continuous and significant enrichment between 21 and 270 days, followed by a steady state (1.6–6.1 mg/L); (iii) the highest oxygen level applied (O60) was associated with a significant increase between 60 and 270 days, followed by a steady state (2.6–5.6 mg/L); (iv) without oxygen supply (N) there was a significant increase between 8 and 60 days, and between 60 and 270 days.

The different evolution patterns, especially after 270 days of ageing, can be assigned to the balance between the following phenomena: oxidation of coniferaldehyde from the wood and resulting from lignin’s hydroalcoholysis [46,53] and possibly the hydrolysis of a galloylglucoside precursor [vanillin-(6′-*O*-galloyl)-*Ο*-β-d-glucopyranoside] recently found in WSs aged in oak wood [57] (additive) giving rise to vanillin; vanillin oxidation, giving rise to vanillic acid [13,53], and other reactions [13] (subtractive). According to Viriot et al. [45], vanillin is prone to oxidation during the ageing of WS due to its guaiacyl-type structure. The results suggest that the lowest level of oxygen applied (O15) limited oxidation and other reactions involving vanillin whereas the highest one (O60) boosted them. For this reason, higher final content and lower final content were achieved in these WSs, respectively. An intermediate behaviour was observed in the WS from O30 modality. Iron probably played a role in these reactions.

Furthermore, since vanillin is closely related to the ‘vanilla’ aroma of the aged WSs, the changes observed according to the MOX level, as well as the possible formation of related volatile compounds, such as ethyl vanillate [58], may have a relevant impact on the WSs’ flavour.

##### Syringaldehyde

According to the ANOVA results for syringaldehyde content (Table 3), in each sampling time no significant differences between the WSs aged through different modalities were found. However, the WS O15 showed slightly higher content of this phenolic aldehyde at 21, 60, 180 and 270 days, which ranged from 3.9 to 16.0 mg/L. The WS O30 had slightly lower content at 60, 180, 270 and 365 days, varying between 6.2 and 14.3 mg/L. The WS O60 presented an intermediate content at 60, 180 and 270 days (6.5–14.3 mg/L), but the highest one at 365 days, reaching 24 mg/L. The control WS (N) had the lowest syringaldehyde content at all sampling times, ranging between 1.8 and 13 mg/L.

The syringaldehyde kinetics was similar to that of vanillin in the different ageing modalities until 270 days, as formerly observed in a study on alternative ageing using wood staves but without MOX [46], which is explained by the similar aromatic ring backbone type of both aldehydes. Thereafter, the WS subject to the highest level of oxygen supplied (O60) behaved differently, with a marked increase in the syringaldehyde concentration. This specific pattern can be ascribed to the syringyl structure of syringaldehyde that confers lower sensitivity to oxidation than the guaiacyl structure of vanillin [45]. Indeed, despite the more favourable oxidative conditions existing in the WS O60 in the last ageing phase (Figure 4), the sharp increment observed (c.a. 10 mg/L) points to the large prevalence of additive phenomena over the subtractive ones: the oxidation of sinapaldehyde from the wood and resulting from lignin’s hydroalcoholysis giving rise to syringaldehyde [46,53] *versus* the oxidation of syringaldehyde, giving rise to syringic acid [13,53], and its involvement in other reactions. The slightly lower content of syringic acid found in this WS than in the WS O15 at 365 days (Table 2) corroborates this explanation.

##### Coniferaldehyde

The statistical outcomes shown in Table 3 demonstrate that coniferaldehyde and syringaldehyde behave similarly in the WSs from the studied modalities at different sampling times. In addition, minimal differences between the WSs were observed up to 270 days: O15—2.3–5.1 mg/L; O30—3.5–4.9 mg/L; O60—3.6–5.1 mg/L. From 270 to 365 days, this trend remained in the WSs O15 and O30 in which the coniferaldehyde concentration reached 5.7 and 5.5 mg/L, respectively, while in the WS O60 a substantial enrichment in this aldehyde occurred, reaching 8.9 mg/L. Specific evolution was also exhibited by the control WS (N), whose content was similar to that of WS O30 (0.9–5.4 mg/L). The results obtained until 270 days seem to indicate the small influence of oxygen content on the behaviour of this compound. However, the sudden increase of coniferaldehyde concentration in the WS O60 at the end of ageing leads to questioning this hypothesis. Since the same behaviour was observed in the WSs from the two replicates of O60 modality, a problem in their sampling should be excluded. Taking into account the findings of García-Estévez et al. [47], it may have been due to an indirect involvement of oxygen in coniferaldehyde formation. Indeed, the oxidation products of the pool of wood-derived phenolic compounds may have favoured such pathway in this more advanced phase of ageing, in which more reactions took place and give rise to a greater concentration and diversity of compounds, contributing to increase the reactivity of the medium. Nevertheless, its origin from lignin’s hydroalcoholysis in such conditions [53] should also be considered [13,53]. On the other hand, subtractive phenomena may also have occurred due to the sensitivity of this guaiacyl-type aldehyde towards oxidation [45], generating the corresponding benzoic aldehyde (vanillin) and ferulic acid [13,53], although to a lesser extent.

##### Sinapaldehyde

The behaviour of sinapaldehyde in WSs from different ageing modalities and over the ageing period was identical to that of coniferaldehyde (Table 3). This result is in accordance with those reported by García-Estévez et al. [47] in model-solution under different oxygenation levels. The following content’s variation was observed up to 270 days: O15—9.2–22.9 mg/L; O30—13.6–20.4 mg/L; O60—15.2–22 mg/L. From 270 to 365 days, the trend remained in the WSs O15 and O30 in which the coniferaldehyde concentration reached 27.5 and 24.6 mg/L, respectively, while in WS O60 a strong increase took place, reaching 40 mg/L. The content of this aldehyde in the control WS (N) was also closer to that of the O30, varying between 3.3 and 24 mg/L.

The explanation for the sinapaldehyde behaviour in the WS O60 at the end of ageing resembles that of coniferaldehyde. In this case, the oxidation of sinapaldehyde would have been less extensive than that of coniferaldehyde due to the cinnamic nature and syringyl structure of the former [45]; this kind of reaction gave rise to the corresponding benzoic aldehyde, syringaldehyde [13,53].

#### 2.1.4. Furanic Aldehydes

Furanic aldehydes are heterocyclic compounds that contain a furan ring. Some of them (furfural, 5-hydroxymethylfurfural and 5-methylfurfural) exist in very low concentration in chestnut and oak wood [33], but appreciable amounts are accrued as a result of hemicelluloses and cellulose degradation during heat treatment [56], which can later be extracted by the WS during ageing.

The one-way ANOVA results for the WSs’ furanic aldehydes contents during the 365 days of ageing are presented in Table 4. They show that only furfural was detected and quantified in the wine distillate (4.3 mg/L). This compound is formed thermally by degradation of wine pentoses during the distillation process [59]. In addition, furfural results from hemicelluloses, which are more thermosensitive and then preferentially degraded than cellulose (the main source of 5-hydroxymethylfurfural and 5-methylfurfural) during the heat treatment in cooperage [56]. These reasons naturally concurred to make furfural quantitatively more important than 5-hydroxymethylfurfural and 5-methylfurfural in the analysed aged WSs. This outcome is in line with those of previous studies on WSs aged in barrel [60] and by alternative technology without MOX [46] and with MOX using a single flow of oxygen [61].

##### Furfural

The results (Table 4) show no significant differences in the furfural content between WSs aged by the different ageing modalities at each sampling time. Among those aged with MOX, greater similarity was found between O15 and O60 from 21 days on, in which the furfural content varied from 61–74 mg/L and 58–74 mg/L, respectively. The WS O30 presented slightly lower content, ranging from 52 to 68 mg/L. The control WS (N) had the lowest content of this furanic aldehyde throughout ageing (4.3–63.6 mg/L).

The evolution pattern of furfural over time is quite different from that of other studied compounds. Indeed, a very sharp and significant increase occurred in all modalities in the beginning of ageing, followed by a steady state after 21 days in WS O15 and after 60 days in WS O30 and WS O60. A steady state was also observed in the control modality (N) after 60 days of ageing.

Such behaviour reflects greater influence of wood extraction than oxidation and other subtractive reactions on the concentration of this compound in WSs. Besides, despite the involvement of oxygen in the extraction of wood compounds [47], resembling levels were found for furfural in the WSs under less and greater oxygenation (O15 and O60, respectively). For these reasons, it is reasonable to accept that oxygen level (and oxidation reactions) was not a determining factor for the evolution of this furanic aldehyde in the aged WSs. It is noteworthy the similar evolution of furfural attained in WS aged in barrels of different wood species [46,60], with presumably different oxygen transfer rate [35], as well as in WS aged with staves without MOX [46]. On the other hand, other subtractive phenomena, such as those identified in red wine involving phenolic compounds and furfural [62] may have occurred, although to a small extent.

Furfural is closely related to the ‘dried fruits’ aroma of the aged WSs [5]. However, taking into account the aforementioned, no noticeable differences are expected in this attribute of WSs according to the MOX modality.

##### 5-Hydroxymethylfurfural

At each sampling time, the 5-hydroxymethylfurfural content of the WS was not significantly influenced by the ageing modality (Table 4). Even so, the WS from O15 modality presented a slightly higher content of this aldehyde than the other WSs between 21 days and 365 days (14–34 mg/L). Conversely, the WS from O60 modality showed the lowest content from 60 to 365 days (19.5–26 mg/L). An intermediate content was found in the WS from O30 modality between 60 and 365 days (20–30 mg/L), as well as in the control WS (N) from 8 days on (4.0–29 mg/L).

The evolution of 5-hydroxymethylfurfural over time was not significant in the WS O15. The other MOX modalities induced a significant increase from 60 to 180 days, followed by a steady state.

These results suggest that this furanic aldehyde was modulated by the oxygen level. Its accumulation was favoured in a less oxidative medium (O15), while the modality corresponding to a more oxidative medium (O60) showed weaker performance. The oxidation and the involvement of 5-hydroxymethylfurfural in other reactions [62] under more oxidative conditions may explain the differences observed. 

Since 5-hydroxymethylfurfural is closely related to the ‘caramel’ aroma of the aged WSs [5], the changes observed according to the MOX level, may influence the WSs’ flavour.

##### 5-Methylfurfural

The content of 5-methylfurfural of the WSs was not affected by the ageing modalities at each sampling time (Table 4). Besides, comparing the MOX modalities, an inconsistent behaviour was found: the WS O60 presented slightly higher content up to 60 days, followed by the WSs O30 and O15; the WS O30 showed slightly higher content at 180 days, followed by the WSs O15 and O60; the WS O15 had slightly higher content at 270 days, followed by the WSs O30 and O60; the WSs O15 and O30 had the same content, which was slightly higher than that of WS O60 at 365 days. 

Examining the evolution over the time in each ageing modality, significant increase was only found between 180 and 270 days for the WSs O15 and O30, and between 180 and 365 days for the WS O60. No significant variation occurred in the control WS (N). The 5-methylfurfural content varied as follows: 0.1–2.0 mg/L in O15; 0.36–1.97 mg/L in O30; 0.5–1.7 mg/L in O60; 0.09–1.4 mg/L in N.

The results obtained do not allow drawing conclusions about the influence of oxygen on the behaviour of this compound.

In summary, it is interesting to note that, despite the distinct kinetics of the mineral elements and low molecular weight compounds and the differences between the ageing modalities, a more differentiated behaviour after 270 days of ageing was observed. This period had underlying more dissolved oxygen for different WSs (Figure 4) as well as a more reactive medium due to the enrichment in wood-derived compounds and related ones, according to the MOX modalities practiced, and probably their most effective action on the chemical phenomena involving the studied analytes.

### 2.2. Global Behaviour of Mineral Elements and Low Molecular Weight Compounds 

To complement the individual approach based on the ANOVA results, a global analysis was performed using cluster heat maps to understand the behaviour of the analytes as a whole in relation to the ageing modalities, and to find out coherent patterns between them.

The clustering analysis and the corresponding heat maps are the most widely used of all bioinformatics, covering areas as diverse as genetics, medicine, geography and, more recently, food chemistry [63,64]. This graphical representation has been successfully used to assess moderately large data matrices because it is visually stimulating and easily interpreted [63].

**Figure 5:**
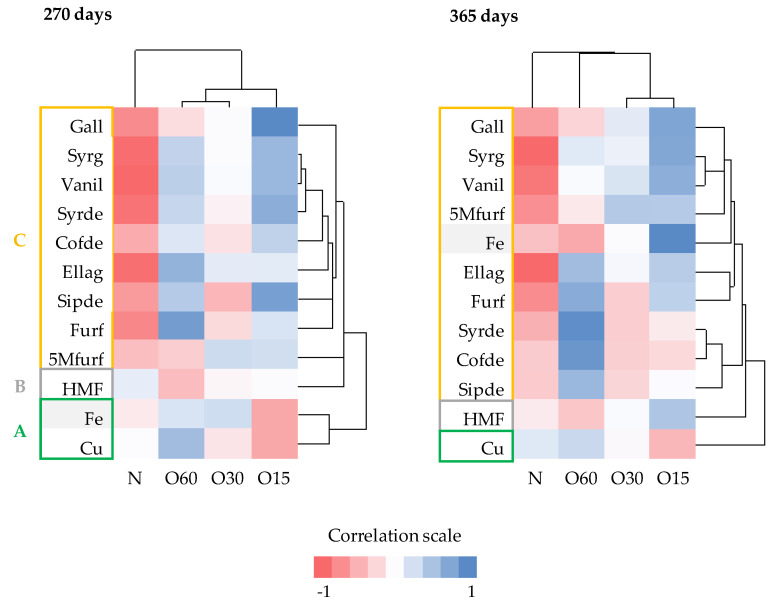
Heat maps plotting clusters of low molecular weight compounds, iron and copper and ageing modalities of wine spirits at 270 and 365 days of ageing.

The heat maps (Figure 5) were generated from the content of mineral elements and low molecular weight compounds in WSs at 270 days and 365 days, which correspond to the most discriminating ageing phase identified above. Blue colours depict a positive correlation between the analytes levels and the ageing modalities while rose colours depict a negative correlation between them.

Both heat maps clustered the WSs into two groups according to the ageing modalities: the first group comprised the control one (N) and the second group contained those from the MOX modalities (O15, O30 and O60). This result highlights the different oxidative media created and confirms the remarkable effect of oxygen on the aged wine spirit’s chemical composition.

Besides, both heat maps clustered the analytes into two major clusters and three subclusters A, B and C.

Subcluster A included copper. This mineral element exhibited a weak positive correlation with the control modality (N) and a strong negative correlation with the less oxidative medium created by the O15 modality as opposed to the low molecular weight compounds. These results corroborate the ANOVA ones. Evidence is also provided about the weak relationship between Cu and the studied compounds via chemical phenomena underlying ageing. Iron belonged to this subcluster at 270 days but changed to subcluster C at 365 days; its intervention in reactions involving gallic acid, syringic acid, vanillin (with a closer relationship with iron within subcluster C) and also ellagic acid at the end of ageing is a plausible reason for the observed cluster.

Subcluster B comprised only the 5-hydroxymethylfurfural owing to its negative correlation with the most oxidative medium created by the O60 modality. Weak correlations with the other ageing modalities at 270 days and a noticeable increase in the positive correlation with O15 at 365 days were displayed. These results reinforce those of ANOVA and demonstrate the specific behaviour of 5-hydroxymethylfurfural among the studied low molecular weight compounds.

Subcluster C included the other low molecular weight compounds. These analytes shared a strong negative correlation with the control modality (N), which points out that the oxygen supplied (in addition to that released by the wood) was needed for their accumulation and evolution in the WS. Attention should be devoted to some restricted groups existing in this subcluster: (i) the group formed exclusively by gallic acid, exhibiting the highest positive correlation with O15 and a weak negative correlation with O60, confirming the ANOVA results and defining an indirect relationship between this phenolic acid and the other compounds of this subcluster through the cascade of reactions triggered by its oxidation; (ii) the group consisting of syringic acid and vanillin, with the closest relationship, reflecting a very similar behaviour in the different oxidative media in terms of oxidation and other reactions occurred; syringaldehyde was closely related to this group at 270 days, likely by similar aromatic ring backbone type shared with vanillin and as a precursor of syringic acid via oxidation, but its behaviour changed drastically at 365 days leading to join the coniferaldehyde and sinapaldehyde group, probably due to the lower sensitivity towards oxidation conferred by its syringyl structure; (iii) the group of coniferaldehyde and sinapaldehyde, whose correlation with the most oxidative medium (O60) sharply increased from 270 to 365 days and the positive correlation with the less oxidative medium (O15) at 270 days was replaced by a negative correlation at 365 days, revealing the similarity of these aldehydes’ behaviour, whose cinnamic nature confers them higher resistance to oxidation.

## 3. Materials and Methods 

### 3.1. Experimental Design and Aged WSs Sampling

The experiment was conductedin pilot scale, in 50 L glass demijohns, comprising four ageing modalities: three MOX modalities and one modality with nitrogen application (control), with two replicates. Each demijohn was closed with a proper silicon bung (made by J. M. Gonçalves cooperage, Palaçoulo, Portugal), which, besides ensuring tightness, had one hole with a tight-sealing diffuser tube (for oxygen or nitrogen application) and another hole with a tight-sealing glass tube (up to half the height of the demijohn) for sample collection and dissolved oxygen measurement.

Portuguese chestnut (*Castanea sativa* Mill.) staves were used in all modalities. The staves (50 cm length × 5 cm width × 1.8 cm thickness) were manufactured by J. M. Gonçalves cooperage (Palaçoulo, Portugal) with medium plus toasting level (90 min at an average temperature of 240 °C; 1.8 cm of toasting thickness). The staves were heated in an industrial oven under controled temperature to assure the same toasting level. The quantity of staves inserted into the demijohns was calculated in order to reproduce the surface area to volume ratio of a 250 L barrel (85 cm^2^/L), which is the most commom barrel dimension used in the ageing of WSs.

A wine distillate (alcohol strength, 78.3 *v*/*v*; pH, 5.33; total acidity, as acetic acid, 0.12 g/L of absolute ethanol; volatile acidity, as acetic acid, 0.09 g/L of absolute ethanol) produced by Adega Cooperativa da Lourinhã (Lourinhã, Portugal) through column distillation, was used to fill the demijohns, which were placed in the cellar of Adega Cooperativa da Lourinhã in similar environmental conditions.

MOX was applied to the WS during the ageing period, supplying pure oxygen (X50S Food, Gasin, Portugal) through a multiple diffuser micro-oxygenator (VISIO 6, Vivelys, France) with ceramic diffusers, at different flow rates according to the ageing modality (O). Flow rates were selected based on the results of our previous study [4] and taking into account the following criteria: adequate oxygen supply (avoiding excessive oxidation); possibility of shortening the ageing period and reducing the ageing’s costs to achieve a sustainable ageing technology. The micro-oxygenator, of volumetric type, was adapted by Vivelys to provide the intended low flow rates in the 50 L demijohns. In addition, pure nitrogen (X50S Food, Gasin, Portugal) was applied continuously over the ageing time through a specific device (Gasin, Portugal) to the WS in one of the ageing modalities (N) in order to decrease the dissolved oxygen as much as possible, acting as a control.

Description of the ageing modalities and the corresponding samples is shown in Table 5.

According to previous results [34], the kinetics of wood-derived compounds under an oxygen flow rate of 2 mL/L/month, applied by MOX, tended to a steady state after 12 months of ageing. Thus, the ageing period in the present trial was set at 365 days. At 8, 21, 60, 180, 270 and 365 days of ageing, after shaking the demijohns, the eight aged WSs (O151, O152, O301, O302, O601, O602, N1 and N2) were sampled, at middle height of the demijohn (through the glass tube placed in the silicone bung), and analysed; a total of 48 samples were taken.

### 3.2. Reagents

Ellagic acid dehydrate (Ellag), gallic acid monohydrate (Gall), vanillin (Vanil), 5-hydroxymethylfurfural (HMF), furfural (furf) and 5-methylfurfural (5mfurf) were purchased from Fluka (Buchs, Switzerland). Syringaldehyde (Syrde), coniferaldehyde (Cofde), sinapaldehyde (Sipde), and 4-hydroxybenzaldehyde were purchased from Aldrich (Steinheim, Germany). All of them were used as standards (purity > 97%) without further purification. The standard solutions were freshly prepared prior to use with ethanol/water (75:25 *v*/*v*). All solvents used in the chromatographic analysis were HPLC gradient grade purchased from Merck (Darmstadt, Germany). For mineral analysis, monoelement standard solutions of Fe and Cu, 1000 mg/L (Perkin Elmer, Norwalk, USA), and ethanol reagent grade 99.9%, were used. Nitric acid reagent grade was double-distilled using an infra-red subboiling distillatory system (model BSB-939-IR, Berggof, Germany). Purified water (conductivity < 0.1 µS/cm) was produced using a Arium Comfort apparatus (Sartorius, Göttingen, Germany).

### 3.3. Iron and Copper Analysis

The WS samples were analyzed in terms of their Fe and Cu contents by atomic absorption spectrophotometry using a Flame Atomic Absorption Spectrophotometer - FAAS (Perkin Elmer, Analyst 100), equipped with an air-acetylene burner and hollow-cathode lamps (Perkin-Elmer Lumina). The experimental conditions have been described previously in detail [19]. Analytical calibration was established with aqueous standard solutions (in 70% *v*/*v* etanol; 1% *v*/*v* nitric acid): 0.25, 0.50, 1.00, 2.50 mg/L (Fe) and 0.5, 1.0, 2.0, 4.0 mg/L (Cu). All the WS samples were clarified by centrifugation (7.000 *g*, 10 min, 20 °C) and directly analysed, in triplicate. Total Fe and Cu concentrations were obtained by interpolation of the corresponding calibration curves.

### 3.4. Low Molecular Weight Compounds Analysis

Among the low molecular weight compounds quantified by HPLC in the wine distillate and in the aged WSs, 10 target compounds were selected (phenolic acids - ellagic acid, gallic acid, syringic acid; phenolic aldehydes—vanillin, syringaldehyde, coniferaldehyde, sinapaldehyde; furanic aldehydes—5-hydroxymethylfurfural, furfural, 5-methylfurfural). Selection was based on their ability, revealed in previous studies, to differentiate this kind of spirit drinks [2]. Quantification was performed as described by Canas et al. [65], using a HPLC Lachrom Merck Hitachi system (Merck, Darmstadt, Germany) equipped with a quaternary pump L-7100, a column oven L-7350, a UV-Vis detector L-7400, and an autosampler L-7250, and coupled to HSM D-7000 software (Merck, Darmstadt, Germany) for management, acquisition and treatment of data. A 250 mm × 4 mm i.d. LiChrospher RP 18 (5 μm) column (Merck, Darmstadt, Germany) was used as stationary phase. Detection was made at 280 nm for furanic aldehydes and phenolic acids, and 320 nm for phenolic aldehydes. Samples were spiked with an internal standard (20 mg/L of 4-hydroxybenzaldehyde), filtered through 0.45 μm membrane (Filter-Bio, Nantong, China) and analysed by direct injection of 20 μL. The analysis was performed in duplicate. Quantification of each compound by HPLC was carried out through a calibration curve made with the corresponding commercial standard. The identification of chromatographic peaks was made by the retention time, UV-Vis spectra matching with standards, and was also confirmed by LC-MS. The chromatographic purity of the peaks and the UV-Vis spectra (200–400 nm) were performed using a Waters system equipped with a photodiode-array detector (Waters 996), with the same chromatographic conditions, managed by ‘Millennium 2010’ software (Waters, Milford, MA, USA). The LC-MS analysis was carried out on a Dionex Ultimate 3000/ThermoFisher Scientific system with similar chromatographic conditions. Full-scan MS data was acquired over an m/z range of 100–1000 with acquisition rate of 3 Hz. Other MS conditions were: ion spray voltage, ±4.9 kV; capillary voltage, 20 V and −60 V; RF loading, 90%; nebulizing gas (nitrogen) pressure, 35 psi; drying gas (nitrogen) pressure, 10 psi; drying gas temperature, 350 °C. Data acquisition and analysis were performed using the Xcalibur 2.2 software (ThermoFisher Scientific, Waltham, MA, USA).

### 3.5. Dissolved Oxygen Measurement

The dissolved oxygen (DO) of the WSs was monitored in situ through discrete measurements during the ageing period—at 0 (wine distillate), 8, 15, 21, 30 days and then monthly until 365 days—using a phase fluorometer (NEOFOX KIT PROBE, Ocean Optics, Dunedin, FL, USA) with a FOSPOR-R sensor probe with a lowest level detection of 4 μg/L (at 1 atm); measurement was performed on a water-based basis. Measurement was made at middle height of the demijohn through the glass tube placed in the silicone bung.

### 3.6. Statistical Analysis

A one-way analysis of variance (ANOVA) was performed to examine the effect of the oxygenation level (O15, O30, O60 and N), as a fixed factor, on the iron, copper and low molecular weight compounds contents of the aged WSs in each sampling time. Another one-way ANOVA was carried out to assess the significance of mineral elements and compounds’ kinetics over the ageing time. Fisher’s least significant difference (LSD) test was made to compare the averages when a significant difference (*p* < 0.05) was detected. All the calculations were carried out using Statistica version 7.0 software (Statsoft Inc., Tulsa, OK, USA).

For heat maps analysis, firstly, the r value of Pearson correlation was determined between modalities and low molecular weight compounds, iron and copper. The obtained r values were grouped using the clustering analysis. For this analysis, Statistica version 7.0 software (StatSoft Inc., Tulsa, OK, USA) was used. On the heatmaps, blue colours depict a positive r and rose colours depict a negative r.

## 4. Conclusions

This study provides innovative information on the influence of MOX level together with chestnut wood on the behaviour of low molecular weight compounds, iron and copper during 365 days of WS’s ageing, thus contributing to a better understanding of the WS ageing chemistry.

Most of the low molecular weight compounds studied underwent significant content variation over the time and revealed different behaviour according to the oxygenation level of the WS. Gallic acid, syringic acid and vanillin showed higher contents over time in the WS aged under mild oxidative conditions (MOX: 15 days at 2 mL/L/month followed by 0.6 mL/L/month until 365 days), which reflected their sensitivity towards oxidation. Among them, syringic acid and vanillin presented the closest behaviour. Ellagic acid, syringaldehyde, coniferaldehyde and sinapaldehyde exhibited an opposite pattern, with higher contents in the WS aged under stronger oxidative conditions (MOX: 60 days at 2 mL/L/month followed by 0.6 mL/L/month until 365 days), especially during the last ageing phase (270–365 days). Among them, a closer relationship between coniferaldehyde and sinapaldehyde was found. Besides, intermediate contents of all compounds were associated with the intermediate oxidative conditions (MOX: 30 days at 2 mL/L/month followed by 0.6 mL/L/month until 365 days). The lowest contents of these compounds were observed in the control WS. These results demonstrated the pivotal role of oxygen on the WS ageing, either supplied by MOX (replicating the oxygen transfer occurring in the barrel) or released from the wood itself (shown by the control modality). Oxygen seems to have modulated the compounds’ kinetics by influencing the additive phenomena, including their extraction from the wood and reactions involving their precursors in the liquid medium, and the subtractive phenomena, such as oxidation reactions and subsequent interactions with other compounds. It is noteworthy that a higher level of accumulated oxygen, as well as a more reactive medium resulting from the enrichment of WS in wood-derived and related compounds, may have promoted a more differentiated behaviour of the analytes between the ageing modalities after 270 days. The furanic aldehydes (furfural, 5-hydroxymethylfurfural and 5-methylfurfural) presented specific behaviours, which require further investigation.

Regarding the mineral composition, iron and copper total contents were low and also underwent significant variation over the ageing time; an increase up to 270 days followed by a decrease was exhibited by iron, while the opposite behaviour was shown by copper. A weak correlation between copper and the studied compounds was evidenced whereas a close relationship between iron, gallic acid, syringic acid, vanillin and ellagic acid at the end of the ageing period was found, suggesting the intervention of this mineral element in oxidation and other reactions involving such compounds. In future studies, the different physicochemical forms of the mineral elements (speciation analysis) that together constitute the total concentrations, and the balance of their different oxidation forms (redox speciation) should be assessed for a better understanding of their kinetics and risk of haze formation, among others. Furthermore, investigation on the relationship between the metal oxidation forms and WS compounds (e.g., ethanol, phenolics) in a model WS system, would provide a comprehensive insight into the reactivity of iron and copper. 

## Figures and Tables

**Table 1:** Contents of iron and copper in the aged WSs according to the ageing modalities and the ageing time.

Modality		Time (days)							
		0	8	21	60	180	270	365	
**Fe** (mg/L)	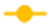 **O15**	0.086 ± 0.002 *a*	0.095 ± 0.003 *ab*	0.095 ± 0.004 *ab*	0.12 ± 0.01 *b*	0.20 ± 0.03 *c*	0.27 ± 0.01 *d*	0.23 ± 0.02 *c*	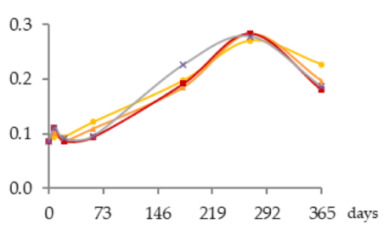
	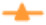 **O30**	0.086 ± 0.002 *a*	0.10 ± 0.01 *ab*	0.087 ± 0.006 *a*	0.109 ± 0.001 *b*	0.185 ± 0.005 *c*	0.28 ± 0.02 *d*	0.20 ± 0.01 *c*	
	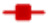 **O60**	0.086 ± 0.002 *a*	0.111 ± 0.005 *a*	0.087 ± 0.004 *a*	0.094 ± 0.003 *a*	0.19 ± 0.03 *b*	0.28 ± 0.03 *c*	0.18 ± 0.02 *b*	
	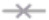 **N**	0.086 ± 0.002 *a*	0.111 ± 0.004 *b*	0.09 ± 0.004 *ab*	0.10 ± 0.01 *ab*	0.23 ± 0.02 *d*	0.279 ± 0.008 *e*	0.186 ± 0.006 *c*	
**Cu** (mg/L)	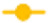 **O15**	0.939 ± 0.009 *f*	0.767 ± 0.004 *e*	0.672 ± 0.006 *d*	0.606 ± 0.016 *c*	0.314 ± 0.020 *a*	0.318 ± 0.002 *a*	0.445 ± 0.003 *b*	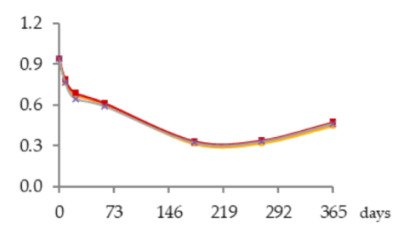
	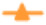 **O30**	0.939 ± 0.009 *f*	0.78 ± 0.03 *e*	0.68 ± 0.02 *d*	0.606 ± 0.004 *c*	0.321 ± 0.005 *a*	0.330 ± 0.009 *a*	0.457 ± 0.002 *b*	
	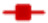 **O60**	0.939 ± 0.009 *f*	0.79 ± 0.004 *e*	0.691 ± 0.016 *d*	0.616 ± 0.027 *c*	0.34 ± 0.01 *a*	0.34 ± 0.02 *a*	0.47 ± 0.03 *b*	
	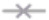 **N**	0.939 ± 0.009 *f*	0.77 ± 0.04 *d*	0.65 ± 0.03 *c*	0.59 ± 0.01 *c*	0.32 ± 0.02 *a*	0.33 ± 0.03 *a*	0.46 ± 0.05 *b*	

Results expressed as mean ± standard deviation. For each element: ageing time (days)—means within the same row followed by different lowercase letters (*a*,*b*,*c*,*d*,*e*,*f*) are significantly different (*p* < 0.05); ageing modality—means within the same column followed by different uppercase letters (A,B) are significantly different (*p* < 0.05); 0 days—corresponds to the wine distillate used to fill the demijohns. Fe—iron; Cu—copper. O15, O30, O60—MOX levels; N—Nitrogen (control).

**Table 2:** Contents of phenolic acids in the aged WSs according to the ageing modalities and the ageing time.

Modality		Time (days)							
		0	8	21	60	180	270	365	
**Gall** (mg/L)	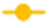 **O15**	nd	23 ± 5 *a*	45 ± 7 *ab*	78 ± 9 *b*	112 ± 18 *c*	123 ± 19 *c*	123 ± 17 *c*	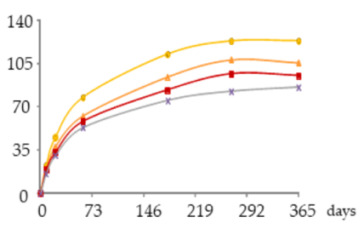
	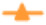 **O30**	nd	19 ± 3 *a*	38 ± 3 *ab*	63 ± 9 *b*	94 ± 11 *c*	108 ± 16 *c*	106 ± 15 *c*	
	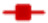 **O60**	nd	19 ± 1 *a*	33 ± 3 *a*	58 ± 4 *b*	83 ± 10 *c*	96 ± 10 *c*	95 ± 13 *c*	
	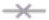 **N**	nd	16 ± 4 *a*	30 ± 6 *ab*	53 ± 10 *bc*	75 ± 15 *c*	82 ± 17 *c*	86 ± 20 *c*	
**Ellag** (mg/L)	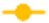 **O15**	nd	2.2 ± 0.2 *a*	4.5 ±0.1 *a*	8.7 ± 0.9 *b*	14 ± 1 *c*	24 ± 2 *d B*	23 ±3 *d*	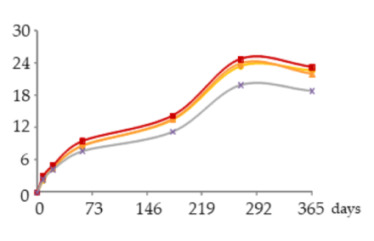
	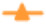 **O30**	nd	2.4 ± 0.2 *a*	4.6 ± 0.1 *b*	8.6 ± 0.3 *c*	14 ± 1 *d*	24 ± 1 *f B*	22 ± 1 *e*	
	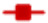 **O60**	nd	3.07 ± 0.07 *a*	5.1 ± 0.4 *b*	9.5 ± 0.4 *c*	14 ± 1 *d*	25 ± 0 *f B*	23 ± 1 *e*	
	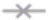 **N**	nd	2.4 ± 0.2 *a*	4.3 ± 0.2 *b*	7.7 ± 0.5 *c*	11 ± 1 *d*	20 ± 0 *e A*	19 ± 1 *e*	
**Syrg** (mg/L)	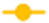 **O15**	nd	1.8 ± 0.0 *a*	3.1 ± 0.4 *b*	5.5 ± 0.7 *c*	9.3 ± 0.5 *d B*	10 ± 0 *d*	13 ± 1 *e B*	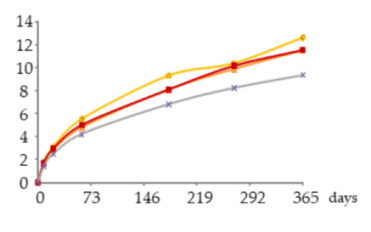
	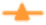 **O30**	nd	1.7 ± 0.1 *a*	2.8 ± 0.1 *b*	4.8 ± 0.2 *c*	8.1±0.3 *d AB*	10 ± 1 *e*	12 ± 0 *f B*	
	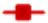 **O60**	nd	1.7 ± 0.1 *a*	2.9 ± 0.0 *b*	5.0 ± 0.1 *c*	8.1±0.1 *d AB*	10 ± 0 *e*	12 ± 0 *f B*	
	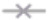 **N**	nd	1.4 ± 0.1 *a*	2.5 ± 0.1 *b*	4.2 ± 0.7 *c*	6.8 ± 0.7 *d A*	8 ± 1 *de*	9 ± 1 *e A*	

Results expressed as mean ± standard deviation. For each element: ageing time (days)—means within the same row followed by different lowercase letters (*a*,*b*,*c*,*d*,*e*,*f*) are significantly different (*p* < 0.05); ageing modality—means within the same column followed by different uppercase letters (*A*,*B*) are significantly different (*p* < 0.05); nd—not detected. 0 days—corresponds to the wine distillate used to fill the demijohns. Gall—gallic acid; Ellag—ellagic acid; Syrg—syringic acid. O15, O30, O60—MOX levels; N—Nitrogen (control).

**Table 3:** Contents of phenolic aldehydes in the aged WSs according to the ageing modalities and the ageing time.

Modality		Time (days)							
		0	8	21	60	180	270	365	
**Vanil** (mg/L)	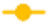 **O15**	nd	0.85 ± 0.02 *a*	1.6 ± 0.1 *b*	2.7 ± 0.0 *c B*	4.1 ± 0.4 *d*	5.7 ± 0.1 *e B*	6.1 ± 0.3 *e*	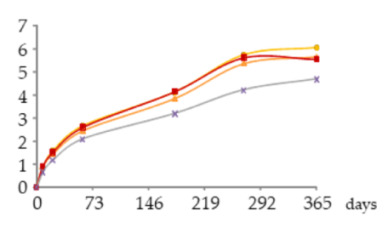
	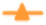 **O30**	nd	0.9 ± 0.1 *a*	1.4 ± 0.1 *b*	2.5 ± 0.1 *c AB*	3.9 ± 0.1 *d*	5.4 ± 0.1 *e B*	5.7 ± 0.0 *f*	
	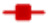 **O60**	nd	0.9 ± 0.0 *a*	1.5 ± 0.1 *a*	2.6 ± 0.0 *b B*	4.2 ± 0.2 *c*	5.6 ± 0.0 *d B*	5.6 ± 0.7 *d*	
	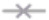 **N**	nd	0.66 ± 0.07 *a*	1.2 ± 0.2 *ab*	2.1 ± 0.3 *b A*	3.2 ± 0.5 *c*	4.2 ± 0.4 *d A*	4.7 ± 0.6 *d*	
**Syrde** (mg/L)	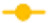 **O15**	nd	2.1 ± 0.0 *a*	3.9 ± 0.1 *b*	6.8 ± 0.1 *c*	11.2 ± 0.7 *d*	14.9 ± 0.0 *e*	16.0 ± 0.2 *f*	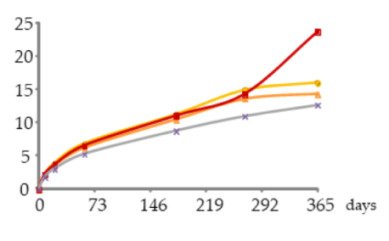
	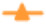 **O30**	nd	2.2 ± 0.2 *a*	3.5 ± 0.1 *b*	6.2 ± 0.1 *c*	10.5 ± 0.2 *d*	13.6 ± 0.5 *e*	14.3 ± 0.4 *f*	
	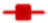 **O60**	nd	2.2 ± 0.1 *a*	3.6 ± 0.0 *a*	6.5 ± 0.1 *a*	11.00 ± 0.07 *a*	14.3 ± 0.2 *a*	24 ± 1 *a*	
	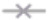 **N**	nd	1.8 ± 0.3 *a*	3.0 ± 0.4 *a*	5.3 ± 0.9 a*b*	8.8 ± 1.6 *bc*	11 ± 2 c*d*	13 ± 2 *d*	
**Cofde** (mg/L)	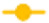 **O15**	nd	1.1 ± 0.1 *a*	2.3 ± 0.2 *b*	3.8 ± 0.3 *c*	5.0 ± 0.6 *d*	5.1 ± 0.4 *d*	5.7 ± 0.5 *d*	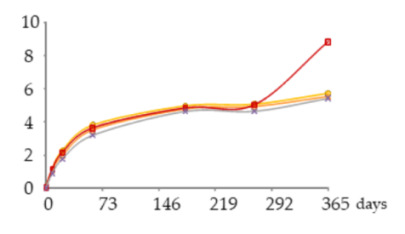
	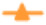 **O30**	nd	1.1 ± 0.1 *a*	2.0 ± 0.2 *b*	3.5 ± 0.3 *c*	4.8 ± 0.2 *d*	4.9 ± 0.2 *d*	5.5 ± 0.3 *e*	
	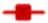 **O60**	nd	1.1 ± 0.1 *a*	2.1 ± 0.4 *a*	3.6 ± 0.5 *a*	4.9 ± 0.7 *a*	5.1 ± 0.7 *a*	8.9 ± 0.6 *a*	
	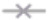 **N**	nd	0.9 ± 0.0 *a*	1.8 ± 0.1 *b*	3.2 ± 0.2 *c*	4.7 ± 0.3 *d*	4.7 ± 0.3 *d*	5.4 ± 0.1 *e*	
**Sipde** (mg/L)	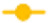 **O15**	nd	4.4 ± 0.0 *a*	9.2 ± 0.3 *b*	16.2 ± 0.4 *c*	23 ± 2 *d*	22.9 ± 0.5 *d*	27.5 ± 0.8 *e*	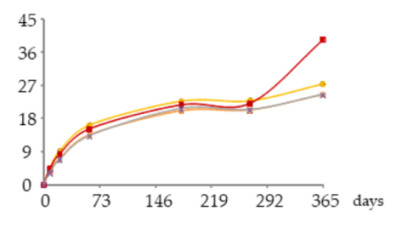
	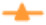 **O30**	nd	3.8 ± 0.2 *a*	7.2 ± 0.3 *b*	13.6 ± 0.5 *c*	20.2 ± 0.0 *d*	20.4 ± 0.0 *d*	24.6 ± 0.2 *e*	
	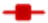 **O60**	nd	4.3 ± 0.2 *a*	8.4 ± 0.6 *a*	15.2 ± 0.7 *a*	22 ± 1 *a*	22 ± 1 *a*	40 ± 2 *a*	
	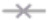 **N**	nd	3.3 ± 0.4 *a*	6.8 ± 1.0 *b*	13 ± 2 *c*	21 ± 2 *d*	20 ± 2 *d*	24 ± 3 *d*	

Results expressed as mean ± standard deviation. For each compound: ageing time (days)—means within the same row followed by different lowercase letters (*a*,*b*,*c*,*d*,*e*,*f*) are significantly different (*p* < 0.05); ageing modality—means within the same column followed by different uppercase letters (*A*,*B*) are significantly different (*p* < 0.05); nd—not detected. 0 days—corresponds to the wine distillate used to fill the demijohns. Vanil—vanillin; Syrde—syringaldehyde; Cofde—coniferaldehyde; Sipde—sinapaldehyde. O15, O30, O60—MOX levels; N—Nitrogen (control).

**Table 4:** Contents of furanic aldehydes in the aged WSs according to the ageing modalities and the ageing time.

Modality		Time (days)							
		0	8	21	60	180	270	365	
**Furf** (mg/L)	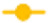 **O15**	4.3 ± 0.2 *a*	33 ± 2 *b*	61 ± 6 *c*	74 ± 9 *c*	74 ± 5 *c*	74 ± 7 *c*	74 ± 7 *c*	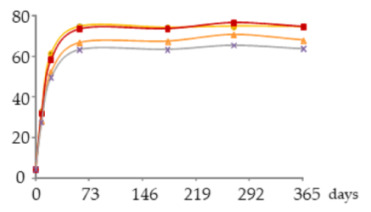
	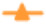 **O30**	4.3 ± 0.2 *a*	28.3 ± 0.2 *b*	52 ± 4 *c*	67 ± 3 *d*	67 ± 4 *d*	70 ± 4 *d*	68 ± 3 *d*	
	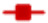 **O60**	4.3 ± 0.2 *a*	31.8 ± 0.9 *b*	58 ± 1 *c*	73 ± 3 *d*	73 ± 4 *d*	76 ± 4 *d*	74 ± 6 *d*	
	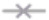 **N**	4.3 ± 0.2 *a*	28 ± 2 *b*	50 ± 5 *c*	63 ± 4 *d*	63 ± 2 *d*	65 ± 2 *d*	63.6 ± 0.4 *d*	
**HMF** (mg/L)	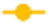 **O15**	nd	6 ± 2 *a*	14 ± 6 *a*	26 ± 12 *a*	32 ± 13 *a*	30 ± 12 *a*	34 ± 14 *a*	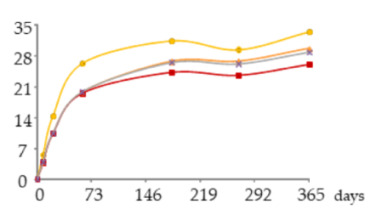
	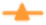 **O30**	nd	3.9 ± 0.3 *a*	10.4 ± 0.3 *a*	20 ± 2 *b*	27 ± 4 *bc*	27 ± 5 *bc*	30 ± 6 *c*	
	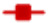 **O60**	nd	3.87 ± 0.08 *a*	10.3 ± 0.8 *b*	19.5 ± 0.8 *c*	24 ± 2 *d*	24 ± 2 *d*	26 ± 3 *d*	
	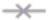 **N**	nd	4.0 ± 0.5 *a*	10.5 ± 0.9 *ab*	20 ± 2 *bc*	27 ± 5 *c*	26 ± 5 *c*	29 ± 7 *c*	
**5Mfurf** (mg/L)	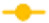 **O15**	nd	0.03 ± 0.02 *a*	0.1 ± 0.1 *a*	0.2 ± 0.1 *a*	0.44 ± 0.07 *a*	1.7 ± 0.3 *b*	2.0 ± 0.3 *b*	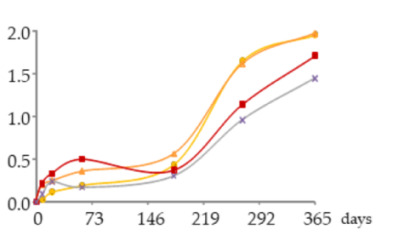
	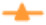 **O30**	nd	0.2 ± 0.0 *a*	0.25 ± 0.03 *a*	0.36 ± 0.08 *a*	0.56 ± 0.06 *a*	1.6 ± 0.4 *b*	1.97 ± 0.07 *b*	
	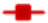 **O60**	nd	0.21 ± 0.06 *a*	0.33 ± 0.04 *a*	0.5 ± 0.2 *a*	0.4 ± 0.1 *a*	1.1 ± 0.9 *ab*	1.7 ± 0.5 *b*	
	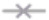 **N**	nd	0.09 ± 0.03 *a*	0.24 ± 0.07 *a*	0.2 ± 0.1 *a*	0.3 ± 0.2 *a*	1.0 ± 0.8 *a*	1.4 ± 0.5 *a*	

Results expressed as mean ± standard deviation. For each compound: ageing time (days)—means within the same row followed by different lowercase letters (*a*,*b*,*c*,*d*) are significantly different (*p* < 0.05); ageing modality—means within the same column followed by different uppercase letters (*A*,*B*) are significantly different (*p* < 0.05); nd—not detected. 0 days—corresponds to the wine distillate used to fill the demijohns. Furf—furfural; HMF—5-hydroxymethylfurfural; 5Mfurf—5-methylfurfural. O15, O30, O60—MOX levels; N—Nitrogen (control).

**Table 5:** Ageing modalities description.

Modality	Samples Code	MOX Flow Rate	N_2_ Flow Rate
O15	O151; O152	2 mL/L/month—0 to 15th day 0.6 mL/L/month—15 to 365th day	-
O30	O301; O302	2 mL/L/month—0 to 30th day 0.6 mL/L/month—30 to 365th day	-
O60	O601; O602	2 mL/L/month—0 to 60th day 0.6 mL/L/month—60 to 365th day	-
N	N1; N2	-	20 mL/L/month

Chestnut wood with medium plus toasting was used in all modalities.
